# Clinical efficacy of breathing training combined with core stability training in chronic nonspecific low back pain

**DOI:** 10.12669/pjms.39.4.6918

**Published:** 2023

**Authors:** Duoduo Yu, Yaming Yu, Qian Peng, Jingting Luo, Xu He

**Affiliations:** 1 Duoduo Yu, Department of Orthopedics, Sichuan Province Orthopedic Hospital, Chengdu 610041 Sichuan, China; 2 Yaming Yu, Department of Orthopedics, Sichuan Province Orthopedic Hospital, Chengdu 610041 Sichuan, China; 3 Qian Peng, Department of Orthopedics, Sichuan Province Orthopedic Hospital, Chengdu 610041 Sichuan, China; 4 Jingting Luo, Department of Orthopedics, Sichuan Province Orthopedic Hospital, Chengdu 610041 Sichuan, China; 5 Xu He Department of Orthopedics, Sichuan Province Orthopedic Hospital, Chengdu 610041 Sichuan, China

**Keywords:** Breathing training, Core stability training, Chronic nonspecific low back pain

## Abstract

**Objective:**

The study aimed to assess the clinical efficacy of breathing training combined with core stability training in chronic nonspecific low back pain (CNLBP).

**Methods:**

This was a retrospective study. Of 60 included patients with CNLBP admitted by the Sichuan Province Orthopedic Hospital between December 2020 and February 2022. Random number table method was used to divide thirty patients to a control group, and the rest 30 to an observation group. The control group received core stability training, while the observation group underwent breathing training in addition to the exact treatment provided for the control group. To assess the utility of breathing and core stability training for CNLBP treatment, intergroup comparisons were made for clinical outcomes, the VAS, SF- 36, and SCODI scores before treatment and at three and seven weeks post-treatment, and static and dynamic low-back muscular endurance before and after treatment.

**Results:**

The observation group had an overall response rate (ORR) of 96.67%, significantly higher than that (73.33%) of the control group (*p<* 0.05). Following the intervention, the VAS and SCODI scores declined in both groups; The SF-36 score was elevated in both groups, and likewise. At the end of treatment, both groups exhibited improved static and dynamic muscular endurance of the low back, and the improvement was significantly more distinct in the observation group (*p<* 0.05).

**Conclusion:**

Compared with core stability training as a sole treatment, breathing training combined with core stability training can yield better outcomes, ameliorate lumbar spine function, relieve pain and enhance low-back muscular endurance in patients with CNLBP.

## INTRODUCTION

Low back pain (LBP) is defined as pain or discomfort localized in the lumbar and sacral regions of the spine, which may radiate to the lower limbs^1^. About 90% of LBP cases have nonspecific low back pain (NLBP),^1^,^2^ a clinical term for the condition without specific anatomic pathology^2^. Nonsurgical therapies represent the first choice of clinical treatment for CNLBP. Studies of the lumbar vertebrae identify lumbar instability and altered biomechanical structures as major causes of CNLBP and summarize typical manifestations of the disease, including lumbar instability due to varying degrees of stabilizing muscle atrophy and decreased contractility or fatigue of lumbar core muscles.^3^ In CNLBP, the capacity of the lungs and diaphragmatic mechanics change with breathing patterns, with evidence showing that approximately 64% of patients with dyspnea are reportedly at risk of CNLBP. This highlights the importance of improving lumbar core stability and proper breathing in the effective treatment of CNLBP.^4^ Core stability training can help stimulate lumbar core muscle contraction and coordination. Breathing training, a commonly used clinical rehabilitation method, can improve lung ventilation and reverse respiratory abnormalities.^5^ On this basis, 60 patients with CNLBP were enrolled to analyze the utility of breathing training combined with core stability training for the treatment of CNLBP.

## METHODS

This was a retrospective study. A total of 60 patients with CNLBP admitted by the Sichuan Province Orthopedic Hospital between December 2020 and February 2022 Random number table method was used to divide were divided into a control group and an observation group (n= 30 each) using a random number table. The observation group was composed of 18 male and 12 female patients aged from 25 to 54 years (mean age: 40.43 ± 8.42 years), with a disease course of six to 13 months (mean disease course: 9.17 ± 2.35 months). The control group had 21 male and nine female patients aged between 30 and 57 years (mean age: 40.63 ± 8.14 years) and suffering from CNLBP for six to 13 months (mean disease course: 9.13 ± 2.26 months). The two groups were comparable with regard to sex, age, and disease course (all *p>* 0.05).

### Ethical Approval:

The study was approved by the Institutional Ethics Committee of Sichuan Province Orthopaedic Hospital (No.:KY2022-029-001; Date: October 19, 2022), and written informed consent was obtained from all participants.

### Inclusion criteria:

- Meeting the criteria for CNLBP set out by the American Physical Therapy Association^6^ and confirmed to have CNLBP through physical examinations and imaging tests after admission;
- Reporting pain in the low back, lumbosacral area, and hips that worsened after physical activities;
- Age > 18 years;
- Suffering from CNLBP for over three months;
- Reporting tenderness in the lumbosacral area but testing negative for the Lasegue’s sign and Bragard’s sign
- Having a VAS score > 3 points;
- Fully aware of the aspects involved in the study and having signed the informed consent form.

### Exclusion criteria:

- Having received CNLBP treatment within the previous month;
- Present with lumbar disc protrusion and/or fractures resulting in backleg and/or dorsolumbar pain;
- Concomitant with osteoporosis and/or cancer;
- Having comorbid structural alterations of lumbar tissues;
- Diagnosed with severe concurrent conditions, such as heart, liver, and/or kidney dysfunction;
- Having comorbidities like cardiovascular disease and hemorrhagic disorders;
- Pregnant women;
- Transfer or withdrawal before the end of the study.

The control group underwent core stability training, and the observation group received the exact core stability training plus breathing training. *Core stability training:* A training program involving five core stability exercises was provided under the direction of a rehabilitation physician, including push-ups, side bridges, glute bridges, alternating reach and kickbacks, and windshield wipers. The core stability training lasted for eight weeks, one session daily, four days per week. *Breathing training:* The observation group also received diaphragmatic breathing training supervised by the same rehabilitation physician. Diaphragmatic breathing training were repeated ten times per session, respectively, two sessions daily, five days per week for eight weeks.

### Outcome measures:

*Clinical outcomes:* Complete response (CR): no evidence of LBP after the intervention, and free movements; very good partial response (VGPR): remarkable lessening of LBP, and no observable limitations of movements; partial response (PR): LBP relief to some degree, and mild limitations of movement; no response (NR): conditions not meeting any of the above criteria. Overall response rate (ORR) = (CR cases + VGPR cases + PR cases) / total cases × 100%.^7^ *Visual analog scale (VAS):* The VAS score, a patient-reported outcome measure, ranges from 0 to 10, with a higher score indicating greater pain intensity (0: no pain; 10: unbearable pain).

### 36-Item Short Form Survey (SF-36):

The SF-36 measures physical functioning and mental health constructs in eight domains on a 100-point scale, and a higher score represents a more favorable health state. *Simplified Chinese **Oswestry disability index (SCODI):*** The questionnaire consists of 10 items, and each item is scored from zero to five, with a higher score defining a more severe disability. *Static and dynamic muscular endurance of the low back:* Static muscular endurance: Prone position on the floor; both ankles maintained at a given angle and hands placed at the back of your head, with the anterior superior iliac spine in the middle; torso elevated off the floor until it is parallel to the floor, without arching or sagging during the move. Timing began as soon as the participant lifted the torso off the floor. Dynamic muscular endurance: Prone position at 30° to the floor, with the anterior superior iliac spine as midline; the torso raised off the floor and the lower body maintained in the same position; both hands placed over chest, back contracted and body straightened before resuming the starting position. The number of repetitions was recorded as the participant repeated 25 times/min.^8^

### Statistical Analysis:

Data processing was performed using SPSS22.0. Enumeration data were expressed by “(n, %)” and analyzed by the χ^2^ test; measurement data were represented by “(*χ̅*±*S*)” and examined by the *t*-test for intergroup comparisons and the repeated measures ANOVA for comparisons of variables at all time points. Significance was set at the level of *p<* 0.05. Diagrams were plotted using the GraphPad Prism eight software program.

## RESULTS

In this study, 60 patients were treated with an ORR of 85.00% (51/60). The ORR was 96.67% in the observation group and 73.33% in the control group, demonstrating a significant difference between the two groups ( *p<* 0.05). Table-I.

**Table-I:** Intergroup comparison of clinical outcomes [n(%)].

Group	*n*	CR	VGPR	PR	NR	ORR
Observation group	30	13(43.33)	11(36.67)	5(16.67)	1(3.33)	29(96.67)
Control group	30	6(20.00)	14(46.67)	3(10.00)	7(23.33)	22(73.33)
χ^2^						4.706
P						0.030

The repeated measures analysis revealed a significant difference between the within- and between-subject effects of time on VAS score ( *p<* 0.05), suggesting changes in VAS score over time and a difference in such changes between the two groups. Despite the insignificant pre-intervention VAS scores ( *p>* 0.05), both groups had decreased VAS scores following the intervention, and the decline was more significant in the observation group ( *p<* 0.05). Table-II.

**Table-II:** Intergroup comparison of VAS scores at different time points(*χ̅*±*S*), pts).

Group	nxs	Pre-treatment	At 3 weeks post-treatment	At 7 weeks post-treatment
Observation group	50	6.37±1.00	3.93±0.58*	1.47±0.78*
Control group	50	6.53±0.90	4.60±0.68	2.40±0.68
F-value		*F_time-point_* = 809.078; *F_between-group_* = 14.534; *F_interaction_* = 6.006		
P-value		*P_time-point_* < 0.001; *P_between-grou__p<_* 0.001; *P_interaction_* = 0.003		

******Note:****** * p< 0.05 when compared with the control group.

According to the repeated measures analysis, there was a significant difference between the within- and between-subject effects of time on SF-36 score ( *p<* 0.05), hinting at variations with time between the two groups. The pre-intervention SF-36 score did not differ significantly between the two groups ( *p>* 0.05), while the post-intervention SF-36 score was heightened in both groups, and the increase was significantly more pronounced in the observation group ( *p<* 0.05). Table-III.

**Table-III:** Intergroup comparison of SF-36 scores at different time points(*χ̅*±*S*), pts).

Group	*n*	Pre-treatment	At 3 weeks post-treatment	At 7 weeks post-treatment
Observation group	50	30.17±5.65	52.43±7.86*	71.30±5.65*
Control group	50	31.37±5.96	45.37±5.38	64.33±5.70
F-value		*F_time-point_* = 1370.614; *F_between-group_* = 10.141; *F_interaction_* = 22.469		
P-value		*P_time-point_* < 0.001; *P_between-group_* = 0.002; *P_interaction_* < 0.001		

******Note:******

* p< 0.05 when compared with the control group.

The repeated measures analysis showed that the within- and between-subject effects of time were significantly different with respect to SCODI score ( *p<* 0.05), revealing varying degrees of changes over time between the two groups. Although the SCODI scores of the two groups were not significant before the intervention ( *p>* 0.05), a decreased score was seen in both groups after the intervention, and the reduction was more significantly more pronounced in the observation group ( *p<* 0.05). Table-III.

**Table-IV:** Intergroup comparison of SCODI scores at different time points (*χ̅*±*S*), pts).

Group	n	Pre-treatment	At 3 weeks post-treatment	At 7 weeks post-treatment
Observation group	50	28.00±6.83	21.40±4.44*	12.43±3.56*
Control group	50	28.40±6.48	24.27±4.10	18.40±4.60
F-value		*F_time-point_* = 196.671; *F_between-group_* = 7.842; *F_interaction_* = 9.284		
P-value		*P_time-point_* < 0.001; *P_between-group_* = 0.007; *P_interaction_* < 0.001		

******Note:******

* p< 0.05 when compared with the control group.

**Fig.1:**
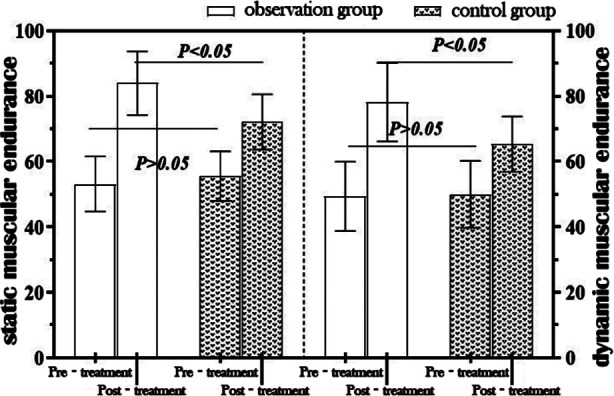
Intergroup comparison of static and dynamic low-back muscular endurance.

Before treatment, static low-back muscular endurance was (53.17 ± 8.45) s for the observation group and (55.57 ± 7.58) s for the control group, while dynamic muscular endurance was (49.37 ± 10.67) and (49.97 ± 10.27) repetitions, respectively. At the end of treatment, enhancement of both static and dynamic muscular endurance was observed in the two groups: (83.97 ± 9.77) s and (78.20 ± 12.09) repetitions for the observation group, and (72.17 ± 8.48) s and (65.20 ± 8.61) repetitions for the control group, demonstrating a significant difference between the two groups (*t* = 4.996 and 4.798, *p<* 0.001). Fig.1.

## DISCUSSION

In this study, compared with the control group, the observation group exhibited sharply decreased VAS and SCODI scores, a markedly elevated SF-36 score, significant improvements in static and dynamic low-back muscular endurance, and a higher ORR of 96.67% at the end of the 8-week breathing and core stability training program. It is believed that breathing training combined with core stability training can substantially ameliorate the symptoms of CNLBP and promote recovery. A probable explanation is that proper breathing can enhance torso stability.

CNLBP is typically manifested as pain in the lumbar and lumbosacral regions and yet has no definitive pathological-anatomical diagnosis.^9^ It has a long disease course, with more than 12 weeks of duration in most cases. Characterized by a long, slow recovery, a high recurrence rate, and intractability, CNLBP can lead to spinal dysfunction and exert a serious impact on everyday life. Although the pathogenesis of CNLBP has not been fully understood, it is generally accepted that reduced lumbar spine stability plays a crucial part in its development and progression.^10^ Lumbar spine stability is maintained by the interaction of the bony structure with skeletons, muscles, connective tissues, and nervous systems. In patients with CNLBP, the typical LBP is associated with the suppression of deep core muscle function, which can result in varying degrees of core muscle atrophy and even lumbar spine instability of the low back.^11^ Therefore, CNLBP treatment should focus on ameliorating lumbar spine instability.

Core stability training aims at stabilization and coordination of core muscles and thus is recognized as an important approach to pertinently strengthen neuromuscular control and enhance torso muscular endurance.^12^,^13^ The core stability training presented in this study helps improve the tensile strength of muscles and joints by enhancing both static and dynamic stability. Li et al.^14^ studied the effects of acupuncture combined with core stability training on CNLBP and discovered that core stability training could strengthen low-back muscular endurance and improve lumbar spine function. These findings are consistent with our observations: at the end of the core stability training program, the control group had reduced VAS and SCODI scores and an increased SF-36 score; at the end of the intervention, the control group showed evident improvements in both static and dynamic low-back muscular endurance. The evidence from this study suggests that patients with CNLBP can benefit from core stability training.^15^

Wong et al.^16^ reported that lumbar spine stability is adversely affected by respiratory abnormalities and respiratory muscle fatigue and thus proposed substituting the combination therapy of core stability training and inspiratory muscle training for stability exercises as monotherapy. In the study of breathing training combined with McKenzie mechanics therapy for CNLBP, Ding et al.^17^ noted that breathing and stability training could ease pain, improve lumbar spine function, and reduce low-back muscle fatigue in patients with CNLBP. In the present study, the observation group received diaphragmatic breathing training that exercises the diaphragms in all directions to avoid imbalances and achieve an all-around improvement in low back function.^18^-^20^

### Limitations of study:

As this study is limited by the modest sample size and the lack of long-term outcomes observation, future research with a larger sample size and long-term prognosis is required to establish the viability of the combination therapy.

## CONCLUSIONS

Breathing training combined with core stability training outperforms the monotherapy of core stability training in treating CNLBP because it can enhance lumbar spine function, reduce pain and improve low-back muscular endurance.

### Authors’ Contributions:

**DY and YY** designed this study and prepared this manuscript, and are responsible and accountable for the accuracy or integrity of the work.

**QP and JL** collected and analyzed clinical data.

**XH:** Data analysis, significantly revised this manuscript, analysis, and interpretation of data and draft the manuscript.
